# Baseline Susceptibility and Cross-Resistance of HearNPV in *Helicoverpa armigera* (Lepidoptera: Noctuidae) in Brazil

**DOI:** 10.3390/insects13090820

**Published:** 2022-09-09

**Authors:** Dionei Schmidt Muraro, Thaini M. Gonçalves, Douglas Amado, Marcelo F. Lima, Holly J. R. Popham, Paula G. Marçon, Celso Omoto

**Affiliations:** 1 Department of Entomology and Acarology, Luiz de Queiroz College of Agriculture, University of São Paulo, Piracicaba 13419-900, Brazil; 2 AgBiTech, Fort Worth, TX 76155, USA

**Keywords:** cotton bollworm, HearNPV, baculovirus, insect resistance management

## Abstract

**Simple Summary:**

Helicoverpa armigera nucleopolyhedrovirus (HearNPV: Baculoviridae: Alphabaculovirus (Armigen^®^)) is a registered insecticide for the management of cotton bollworm, Helicoverpa armigera (Hübner) (Lepidoptera: Noctuidae) in Brazil. We conducted studies of baseline susceptibility of Brazilian populations of H. armigera to HearNPV (Armigen^®^, AgBiTech, Fort Worth, TX, USA) and cross-resistance between HearNPV and insecticides as valuable knowledge in support of integrated pest management and insect resistance management programs.

**Abstract:**

The marked adoption of bioinsecticides in Brazilian agriculture in recent years is, at least partially, explained by the increasingly higher levels of insect pest resistance to synthetic insecticides. In particular, several baculovirus-based products have been registered in the last 5 years, including *Helicoverpa armigera nucleopolyhedrovirus* (HearNPV: *Baculoviridae: Alphabaculovirus* (Armigen^®^)). Understanding the susceptibility of *Helicoverpa armigera* (Hübner) (Lepidoptera: Noctuidae) to HearNPV is an important step toward development of robust Integrated Pest Management (IPM) and Insect Resistance Management programs (IRM) aimed at managing this serious insect pest. In this study, droplet feeding bioassays were used to characterize the baseline susceptibility to HearNPV (Armigen^®^) in *H. armigera* populations collected from major soybean and cotton-growing regions in Brazil. We defined and validated a diagnostic concentration for susceptibility monitoring of *H. armigera* populations to HearNPV. Additionally, cross-resistance between HearNPV and the insecticides flubendiamide and indoxacarb was evaluated by testing HearNPV in a susceptible strain and in resistant strains of *H. armigera* to these insecticides. A low interpopulation variation of *H. armigera* to HearNPV was detected. The LC_50_ values ranged from 1.5 × 10^5^ to 1.1 × 10^6^ occlusion bodies (OBs) per mL (7.3-fold variation). The mortality rate at the identified diagnostic concentration of 6.3 × 10^8^ OBs/mL, based on the calculated LC_99_, ranged from 98.6 to 100% in populations of *H. armigera* collected from 2018 to 2020. No cross-resistance was detected between HearNPV and flubendiamide or indoxacarb. These results suggest that HearNPV (Armigen^®^) can be an effective tool in IPM and IRM programs to control *H. armigera* in Brazil.

## 1. Introduction

The evolution of insect pest resistance to insecticides is one of the main problems in agricultural production systems, worldwide [1]. The cotton bollworm, *Helicoverpa armigera* (Hübner) (Lepidoptera: Noctuidae) is considered an important insect pest in both Old and New World countries [2]. Resistance has already been reported in *H. armigera* to pyrethroids [3,4], spinosyns [5], carbamates [6], diamides [7], oxadiazines [8], Bt proteins [9], among others. As a result, the development of new chemical and biological insecticides with new modes of action is important for IRM programs.

*H. armigera* was first reported in Brazil in 2013, causing damage primarily to soybean (*Glycine max* (L.) Merrill) and cotton (*Gossypium hirsutum* L.) [10,11]. Founder populations in Brazil arrived with alleles conferring resistance to synthetic insecticides such as pyrethroid [4]. Insecticides and genetically modified plants expressing Bt proteins were the main control methods utilized in Brazil [12] because of documented cases of resistance [2,13].

The adoption of effective biological control agents such as baculovirus-based insecticides can delay the onset of pesticide resistance [14]. Potential use of baculoviruses in IPM programs stands out as an important pest management tool due to their high efficacy in pest control, specificity, and selectivity, acting mainly on lepidopteran larvae [12,15,16,17]. To best manage and prolong the longevity of new pest management technologies, it is important to characterize the baseline susceptibility before commercial introduction of an insecticide. These data then allow accurate estimation of a diagnostic concentration for routine resistance monitoring [18,19].

In Brazil, the *Helicoverpa armigera* NPV-based bioinsecticide (HearNPV: *Baculoviridae: Alphabaculovirus*), a new mode-of-action insecticide (Group 31, Insecticide Resistance Action Committee-IRAC) was recently registered to control *H. armigera* [20]. HearNPV acts as host-specific occluded pathogenic viruses that specifically target *H. armigera* larval midgut epithelial columnar cell membranes. During primary infection, occlusion bodies are ingested by the larvae and solubilized by their alkaline midgut environment. This causes virions to be released and pass through the peritrophic membrane and fuse with the microvilli of midgut epithelial cells. The envelope of each virion contains at least nine proteins termed *per os* infectivity factors that form an entry complex that is essential for midgut epithelial cell entry [21,22]. A secondary infection begins after the nucleocapsids travel to the nucleus, where they release the viral genome to initiate self-replication. Progeny viruses are then produced to infect larval tissues and organs, eventually leading to larval death.

There are a few reports of resistance evolution in lepidopteran species to specific alphabaculovirus nucleopolyhedrovirus (NPV) isolates, such as in *Spodoptera frugiperda* to SfMNPV [23] and in *Anticarsia gemmatalis* to AgMNPV [24]. However, SfMNPV presented no cross-resistance to different active ingredients (chlorantraniliprole, chlorpyrifos, lambda-cyhalothrin, spinosad, and teflubenzuron) or to the Bt proteins when tested in Brazilian populations of *S. frugiperda* [16]. No cross-resistance was detected between ChinNPV and chemical insecticides in *Chrysodeixis includens* (Walker) (Lepidoptera: Noctuidae) [25] or between HearNPV and Bt proteins in *H. armigera* and *Helicoverpa punctigera* (Hübner) (Lepidoptera: Noctuidae) [26].

Because of its promising adoption as an important tool for IPM and IRM programs, the objectives of this study were to characterize the baseline susceptibility of field populations of *H. armigera* to HearNPV, develop a diagnostic concentration for resistance monitoring programs and investigate cross-resistance to flubendiamide (IRAC MoA group 28) and indoxacarb (IRAC MoA group 22A).

## 2. Materials and Methods

### 2.1. Insects

**Figure 1:**
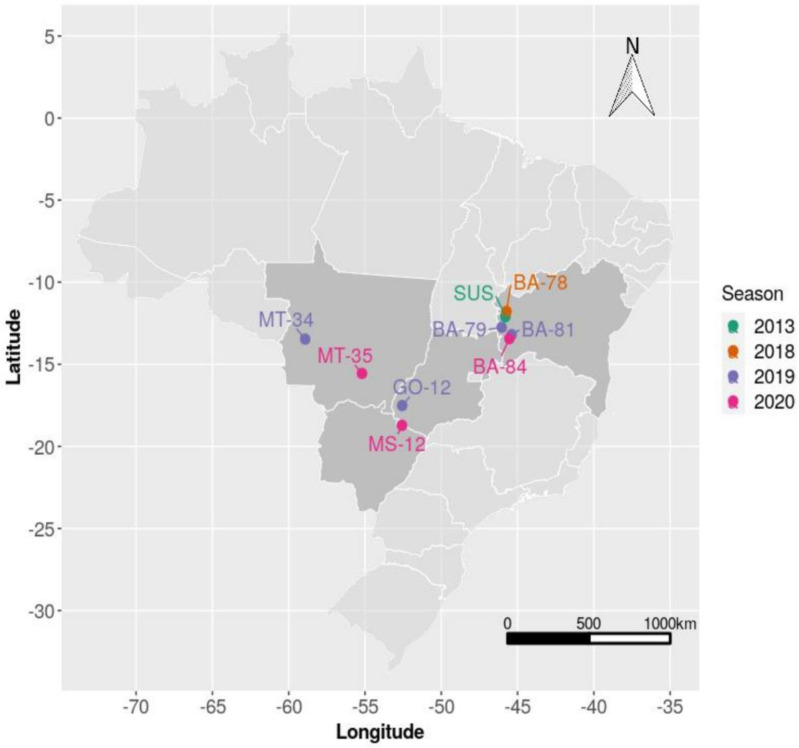
Distribution of *Helicoverpa armigera* populations used to establish baseline susceptibility to HearNPV and validation of a diagnostic concentration.

Field populations of *H. armigera* were collected in major non-Bt soybean and non-Bt cotton growing regions, from 2018 to 2020 crop seasons in Brazil (Table 1; Figure 1). In each location, 800 to 1000 larvae were collected. These field populations were used to characterize the baseline susceptibility and validation of a diagnostic concentration.

For the evaluation of cross-resistance between HearNPV and chemical insecticides, we used strains resistant to flubendiamide (Belt^®^, Bayer Crop Science, Monheim, Germany; 480 g active ingredient (AI)/L) and indoxacarb (Avaunt^®^, FMC, Philadelphia, PA, USA; 150 g AI/L). The strain resistant to flubendiamide (hereafter FBD-R), was selected from a field population collected in Luís Eduardo Magalhães, Bahia, Brazil, (12°05′58″ S and 45°47′54″ W) [7]. The strain resistant to indoxacarb (hereafter AVA-R) was selected from a field population collected in Chapadão do Sul, Mato Grosso do Sul, Brazil, (18°43′29″ S and 52°36′14″ W) [27]. The susceptible laboratory strain (hereafter SUS) was included in all bioassays cited above.

Field populations, resistant, and susceptible strains of *H. armigera* were kept on an artificial diet (adapted from Greene et al. [28]) until pupation. Pupae were transferred to vertical cylindrical cages made of PVC tubes (30 cm high × 25 cm diameter) and covered with tulle netting (egg laying substrate), where adults emerged for mating and oviposition. Each population was composed of 100 pairs per generation, separated in two cages of approximately 50 pairs each. The adult diet consisted of 10% aqueous honey solution offered in moistened cotton balls. The tulle netting with eggs and the honey solution were replaced every 2 days. The eggs were placed in plastic cups (500 mL) and newly hatched larvae (<24 h) were used in bioassays. All populations were maintained in controlled conditions of 25 ± 2 °C, 70% relative humidity and a photoperiod of 14:10 (L:D) h.

### 2.2. Baseline Susceptibility

To characterize the baseline susceptibility of *H. armigera* to the commercial product Armigen^®^ (a.i. HearNPV, concentration 7.5 × 10^9^ occlusion bodies [OBs] per mL), we used six field populations collected in three Brazilian states: Bahia (BA-78, BA-79, and BA-81), Goiás (GO-12), Mato Grosso (MT-34 and MT-35) and a susceptible strain (SUS) (Table 1; Figure 1). Droplet feeding bioassays described by Hughes et al. [29] and Harrison et al. [30] were used to determine viral potency against each population. Seven concentrations of HearNPV, 1 × 10^2^, 1 × 10^3^, 1 × 10^4^, 1 × 10^5^, 1 × 10^6^, 1 × 10^7^, and 1 × 10^8^ OBs/mL, were tested to provide mortality between 5 and 95%. These concentrations were composed of HearNPV diluted in distilled water, 30% sucrose solution, and red dye. Each concentration was applied with an electronic pipette in petri dishes as 0.5 μL droplets. After application, 50 neonates (<24 h old) were placed into each petri dish. Larvae that presented a red color in the midgut after 15 min were determined to have consumed the solution and were then transferred individually into 32-well plastic trays (Advento do Brasil, São Paulo, Brazil) containing the artificial diet [28] without formaldehyde or antibiotics. Trays were then sealed with plastic sheets that allowed air exchange with the external environment, and then placed in a growth chamber at 28 ± 1 °C, 60 ± 10% RH at a photoperiod of 14:10 (L:D) h.

The bioassays were performed in a completely randomized design with 8 to 12 replicates for a total of 64 to 96 neonates tested per concentration, respectively. Mortality was assessed at 1 and 7 days. Death observed in the first day (considered to be death due to the transfer process and not infection) was subtracted from final mortality at 7 days after exposure to HearNPV.

### 2.3. Validation of Diagnostic Concentration

The concentration of 6.3 × 10^8^ OB/mL was estimated from the joint analysis of the entire baseline susceptibility dataset and was used for susceptibility monitoring of *H. armigera* to HearNPV. The methodology previously described was used to validate the diagnostic concentration [29,30]. In these bioassays, 380–550 newly hatched larvae per population were tested. Bioassays were performed with a susceptible strain (SUS) and four field populations collected in different states in Brazil, Bahia (BA-84), Mato Grosso do Sul (MS-12), and Mato Grosso (MT-34 and MT-35) (Table 1; Figure 1).

### 2.4. Cross-Resistance between HearNPV and Insecticides

Resistant strains of *H. armigera* to chemical insecticide (FBD-R and AVA-R) were used to evaluate the cross-resistance pattern with HearNPV-based insecticide. Concentration-response droplet feeding assays were used to characterize the susceptibility of FBD-R, AVA-R, and SUS strains of *H. armigera* to HearNPV. The reference susceptible strain (SUS) was used to compare the 50% lethal concentrations (LC_50_) and calculate resistance ratios. The FBD-R strain showed a resistance ratio of 1770-fold to flubendiamide [7] and the AVA-R strain showed a resistance ratio of 357-fold to indoxacarb [27].

### 2.5. Statistical Analysis

Probit analysis (PROC PROBIT), in SAS ^®^9.1 (SAS Institute 2000, Cary, NC, USA) was used to calculate LC_50_ values and respective 95% confidence intervals (CI) [31]. A likelihood ratio test was conducted to test the hypothesis that the LCp values (lethal concentration at which a percent mortality P is attained) were equal. Pairwise comparisons were performed if the hypothesis was rejected, and significance was declared if CIs did not overlap [32]. Resistance ratios were calculated by dividing the LC_50_ values of resistant strains by the LC_50_ values of the susceptible strain [32]. The diagnostic concentration was estimated from the joint analysis of the entire baseline susceptibility dataset [33]. Mortality data were fitted to a binomial model using the complement log–log link function (PROC PROBIT), in SAS ^®^9.1 (SAS Institute 2000) [31].

## 3. Results

### 3.1. Baseline Susceptibility of H. armigera to HearNPV in Droplet Feeding Bioassays

Field populations and the SUS strain demonstrated similar susceptibility to the HearNPV-based bioinsecticide Armigen^®^ (AgBiTech, Fort Worth, TX, USA). The LC_50_ of *H. armigera* ranged from 1.5 × 10^5^ (MT-35 population) to 1.1 × 10^6^ (SUS strain) OBs/mL (Table 2). These results demonstrate a variation of 7.3-fold in susceptibility among the tested populations of H. armigera. Based on the joint analysis of concentration-mortality data of all populations, the LC_99_ was estimated to be 6.3 × 10^8^ OBs/mL (FL 95% from 2.4 × 10^8^ to 2.3 × 10^9^; n = 2932; slope [±SE] = 0.62 [±0.04]; χ^2^ = 16.21; df = 5). This LC_99_ is the candidate diagnostic concentration for the routine resistance monitoring of *H. armigera* to HearNPV.

### 3.2. Validation of the Candidate Diagnostic Concentration for Resistance Monitoring

The susceptible strain of *H. armigera* (SUS) exposed to the diagnostic concentration of HearNPV (6.3 × 10^8^ OBs/mL) exhibited 98.9% mortality (Table 3). Similar results were observed for four field populations, with mortality ranging from 98.8 to 100%. These results validated the diagnostic concentration of 6.3 × 10^8^ OBs/mL as the rate that causes 99% mortality in HearNPV-susceptible populations. This concentration should be used in routine resistance monitoring programs of *H. armigera* to the HearNPV-based insecticide, Armigen^®^.

### 3.3. Cross-Resistance between HearNPV and Insecticides

The pesticide resistant strains of *H. armigera*, FBD-R and AVA-R, responded similarly to the susceptible strain when exposed to HearNPV (Table 4). The resistance ratios of 0.06 for FBD-R and 1.36 for AVA-R were not significant (Table 3).

## 4. Discussion

The rapid rise of insecticide resistance in *H. armigera* was a result of high selection pressure in soybean, cotton, and maize [20]. All necessary measures must be taken to prevent or delay further increases in the number of cases of resistance. New pest management alternatives and insecticides with new modes of action are fundamental to IPM and IRM. In the present study, we characterized the baseline susceptibility of *H. armigera* field populations to HearNPV and investigated cross-resistance to flubendiamide and to indoxacarb. The field populations of *H. armigera* demonstrated a low variation in susceptibility to HearNPV, with LC_50_ values ranging from 1.5 × 10^5^ to 1.1 × 10^6^ OBs/mL (7.3-fold variation). Similar variation in *H. armigera* susceptibility was observed to different HearNPV isolates, with LC_50_ values ranging from 1.6 × 10^4^ to 3.5 × 10^4^ OBs/mL (2.2-fold variation) [34]. In Brazil, larvae of *S. frugiperda* and *C. includens* were found to have similar variation in susceptibility. The LC_50_ for *S. frugiperda* ranged from 2.2 × 10^6^ to 4.5 × 10^6^ OBs/mL (2.1-fold variation) with SfMNPV [16] and the LC_50_ for *C. includens* ranged from 1.4 × 10^5^ to 7.7 × 10^5^ OBs/mL (5.5-fold variation) with ChinNPV [17]. In contrast, other studies showed a high variation in susceptibility among populations of *S. frugiperda* and *A. gemmatalis*, when exposed to baculovirus-based insecticides [23,24].

A high variation in the susceptibility in *Lymantria dispar* to Lymantria dispar MNPV suggested an antiviral defense that was hormonally controlled [35]. In *H. zea*, the tracheal epidermis became melanized and encapsulated following exposure to *Autographa californica* MNPV, and hemocytes appeared to be resistant to infection and were able to remove virus from the hemolymph [36]. In contrast, the major mechanisms of resistance to indoxacarb in *H. armigera* can be associated with a metabolic detoxification by P450 and carboxyl esterase [37], whereas the most common lepidopteran resistance to flubendiamide are ryanodine receptors target-site mutations [38]. The risk of resistance development is much more likely for a “uni-site” (e.g., flubendiamide and indoxacarb) than for a “multi-site” insecticide or bio-insecticide (e.g., HearNPV) [39].

HearNPV demonstrated high toxicity and low variation in susceptibility among field populations and the susceptible strain of *H. armigera* tested. The low natural variation in HearNPV susceptibility might be related to a high gene flow among populations [13] and founding effects since *H. armigera* is an invasive species [40]. The lack of cross-resistance between the HearNPV-based insecticide and strains resistant to indoxacarb and flubendiamide indicates that Armigen (HearNPV) can be effectively used as a new mode of action insecticide for the control and resistance management of *H. armigera*. Furthermore, the use of insecticides such as indoxacarb and flubendiamide does not promote the selection of resistant individuals to the Armigen^®^ bioinsecticide because there is no cross-resistance between HearNPV and these synthetic insecticides.

A similar lack of cross-resistance between baculovirus and synthetic insecticides has been reported for *S. frugiperda* and *C. includens* [16,25]. In addition, no cross-resistance was reported between Bt proteins and baculovirus in *Plutella xylostella* (L.) (Lepidoptera: Plutellidae) [41]. HearNPV-based baculovirus stands out as a promising tool in the management of insect resistance in a scenario of integration in control strategies seeking to delay the evolution of *H. armigera* resistance to insecticides in Brazil. The strategy of rotating distinct mode of action insecticides is effective if there is no cross-resistance between the control methods used in rotation [42]. Therefore, it is critical to understand the resistance profiles of specific local populations as basis for effective rotation schemes. With this important aspect in mind, other insecticides should be evaluated in future studies.

Results of this study demonstrated that the HearNPV-based insecticide Armigen^®^ may contribute to IPM and IRM programs. Field populations of *H. armigera* tested showed high susceptibility to HearNPV and no cross-resistance to flubendiamide and indoxacarb. For the success of IPM programs that include Armigen, we recommend routine monitoring of the susceptibility of *H. armigera* to HearNPV with the diagnostic concentration proposed in this study. This best practice will allow for early detection of any changes in susceptibility of these populations to HearNPV and adjustment in management tactics accordingly.

We conclude that the biological insecticide HearNPV in Armigen is a feasible tool for control of *H. armigera* field populations in rotation with other mode-of-action insecticides. Baculoviruses co-evolved with their insect hosts and developed very complex host–pathogen interactions, which make it very challenging for the insect pest host to overcome bio-insecticide infection. In addition, the highly specific viral pathogen does not eliminate the entire host population, allowing natural enemies to thrive and further aid in suppressing the target pest [39].

## 5. Conclusions

A low interpopulation variation of *Helicoverpa armigera* to HearNPV was detected in Brazil. No cross-resistance was detected between HearNPV and flubendiamide or indoxacarb. These results suggest that HearNPV (Armigen^®^) can be an effective tool in integrated pest management and insect resistance management programs to control *Helicoverpa armigera* in Brazil.

## Figures and Tables

**Table 1:** Populations of *Helicoverpa armigera* used for the characterization of the baseline susceptibility and validation of the diagnostic concentration to HearNPV.

Population Code	City, State	Host Crop	Latitude (S)	Longitude (W)	Date
SUS	Luís Eduardo Magalhães, BA	Bean	12°05′58″	45°47′54″	September 2013
Season 2018					
BA-78	Luís Eduardo Magalhães, BA	Cotton	11°46′33″	45°43′44″	June 2018
Season 2019					
BA-79	Roda Velha, BA	Soybean	12°45′00″	46°02′25″	December 2018
BA-81	Correntina, BA	Cotton	13°11′34″	45°23′16″	June 2019
GO-12	Mineiros, GO	Soybean	17°30′47″	52°33′48″	December 2018
MT-34	Sapezal, MT	Soybean	13°27’55″	58°55’13″	January 2019
Season 2020					
BA-84	Correntina, BA	Soybean	13°25′55″	45°32′07″	December 2019
MT-35	Campo Verde, MT	Soybean	15°33′29″	55°11′49″	December 2019
MS-12	Chapadão do Sul-MS	Cotton	18°43’13″	52°34’27″	June 2019

**Table 2:** Baseline susceptibility of *Helicoverpa armigera* to HearNPV.

Population	Generation	*n* *^a^*	Slope ± SE *^b^*	LC_50_ (95% CI) *^c^*	χ^2 *d*^	df *^e^*
SUS	F_47_	435	0.48 ± 0.05	1.1 × 10^6^ (3.9 × 10^5^ to 2.6 × 10^6^) a	4.87	4
Season 2018						
BA-78	F_1_	521	0.99 ± 0.16	7.3 × 10^5^ (1.8 × 10^4^ to 4.3 × 10^6^) a	8.55	4
Season 2019						
BA-79	F_1_	486	0.55 ± 0.09	2.5 × 10^5^ (6.5 × 10^3^ to 1.8 × 10^6^) a	9.94	5
BA-81	F_1_	412	0.49 ± 0.12	3.5 × 10^5^ (5.4 × 10^3^ to 1.7 × 10^6^) a	7.68	4
GO-12	F_1_	544	0.51 ± 0.09	4.4 × 10^5^ (1.4 × 10^4^ to 2.9 × 10^6^) a	8.91	4
MT-34	F_1_	543	0.69 ± 0.11	1.9 × 10^5^ (8.6 × 10^4^ to 4.0 × 10^5^) a	7.84	4
Season 2020						
MT-35	F_1_	642	0.53 ± 0.12	1.5 × 10^5^ (6.4 × 10^4^ to 5.1 × 10^5^) a	6.21	4

*^a^* Number of larvae tested. *^b^* Slope and standard error. *^c^* Lethal concentration (OBs/mL) required to kill 50% of neonates in the observation period of 7 days. Values within the column followed by the same letter are not significantly different. *^d^ p* > 0.05 in the goodness-of-fit test. *^e^* Degrees of freedom.

**Table 3:** Mortality of *Helicoverpa armigera* populations at the diagnostic concentration of HearNPV (6.9 × 10^8^ OBs/mL).

Population Code	Generation	Tested	Died	% Mortality (95% CI) *^a^*
SUS	F_47_	450	445	98.9 (97.8–99.5)
BA-84	F_1_	420	415	98.8 (97.8–99.6)
MT-34	F_2_	550	547	99.5 (98.1–99.8)
MT-35	F_2_	380	378	99.6 (98.5–99.8)
MS-12	F_1_	450	450	100.0 (98.7–99.5)

*^a^* Significantly different from each other due to nonoverlap of 95% confidence interval.

**Table 4:** Concentration response of susceptible (SUS), flubendiamide (FBD-R), and indoxacarb (AVA-R) resistant strains of *Helicoverpa armigera* to HearNPV.

Strains	Generation	n ^*a*^	Slope ± SE ^*b*^	LC_50_ (95% CI) ^*c*^	χ^2 *d*^	df ^*e*^	RR ^*f*^
SUS	F_47_	435	0.48 ± 0.05	1.1 × 10^6^ (3.9 × 10^5^–2.6 × 10^6^) a	4.87	4	-
FBD-R	F_34_	521	0.99 ± 0.16	7.3 × 10^4^ (1.8 × 10^3^–4.3 × 10^5^) a	8.55	4	0.06
AVA-R	F_18_	458	0.69 ± 0.11	1.5 × 10^6^ (3.5 × 10^3^–1.4 × 10^6^) a	7.84	4	1.36

^*a*^ Number of larvae tested. ^*b*^ Slope and standard error. ^*c*^ Lethal concentration (OBs/mL) required to kill 50% of neonates in the observation period of 7 days. Values within the column followed by the same letter are not significantly different. ^*d*^ *p* > 0.05 in the goodness-of-fit test. ^*e*^ Degrees of freedom. ^*f*^ Resistance Ratio = LC_50_ of the resistant strains/LC_50_ of the susceptible strain (SUS).

## Data Availability

The data presented in this study are available in article.
